# The importance of muscle activation on the interpretation of muscle mechanical performance

**DOI:** 10.1242/jeb.248051

**Published:** 2024-11-08

**Authors:** Roger W. P. Kissane, Graham N. Askew

**Affiliations:** ^1^ Department of Musculoskeletal Ageing Science, University of Liverpool, The William Henry Duncan Building, 6 West Derby Street, Liverpool L7 8TX, UK; ^2^ School of Biomedical Sciences, University of Leeds, Leeds LS2 9JT, UK

**Keywords:** Work loop, Muscle mechanics, Locomotion, Activation, Net work

## Abstract

The work loop technique was developed to assess muscle performance during cyclical length changes with phasic activation, simulating the *in vivo* conditions of many muscles, particularly during locomotion. To estimate muscle function *in vivo*, the standard approach involves subjecting a muscle to length trajectories and activation timings derived from *in vivo* measurements, whilst simultaneously measuring force. However, the stimulation paradigm typically used, supramaximal, ‘square-wave’ stimulation, does not accurately reflect the graded intensity of activation observed *in vivo*. While the importance of the timing and duration of stimulation within the cycle on estimates of muscle performance has long been established, the importance of graded muscle activation has not been investigated. In this study, we investigated how the activation pattern affects muscle performance by comparing square-wave, supramaximal activation with a graded *in vivo* activation pattern. First, we used *in vivo* electromyography-derived activation patterns and fibre strains from the rabbit digastric muscle during mastication and replayed them *in situ*. Second, we used Hill-type musculoskeletal model-derived activation patterns and fibre strains in a trotting mouse, replayed *ex vivo* in the soleus (SOL) and extensor digitorum longus (EDL) muscles. In the rabbit digastric muscle, square-wave activation led to an 8-fold higher estimate of net power, compared with the *in vivo* graded activation pattern. Similarly, in the mouse SOL and EDL, supramaximal, square-wave activation resulted in significantly greater positive and negative muscle work. These findings highlight that realistic interpretations of *in vivo* muscle function rely upon more accurate representations of muscle activation intensity.

## INTRODUCTION

Muscle properties have been traditionally characterised using isometric and isotonic/isovelocity contractions. Measurements of this type are useful in comparing the relative performance of muscles inter- and intra-specifically (Medler, 2002), and for revealing which intrinsic properties are under selective pressure in relation to performance (Askew, 2023). However, whilst measurements of this type may reveal the limits of performance, they fall short of elucidating muscle function and performance *in vivo* because the conditions under which the measurements are made (e.g. supra-maximally activated, constant muscle length or constant shortening velocity) are rather different from the way in which many muscles operate. For example, muscles typically undergo cyclical length changes and periodic activity during many locomotory behaviours (Askew and Marsh, 2001; Gillis and Biewener, 2001; Gillis et al., 2005).

Skeletal muscles generate force when they are activated, and the load that the muscle acts upon (e.g. inertial, gravitational, elastic, drag, etc.; Marsh, 1999) determines the temporal relationship between muscle length change and force generation and hence the muscle's mechanical performance, i.e. whether the muscle shortens and generates work (concentric contractions), or lengthens and absorbs energy (eccentric contraction). Because the load often varies throughout a length change cycle, the mechanical role of the muscle may be complex and involve both energy absorption and work generation (Ahn and Full, 2002; Eng et al., 2019; Sponberg et al., 2023). The work loop technique was developed to allow muscle performance to be quantified during cyclical length changes (Machin and Pringle, 1959), with phasic activation later incorporated for synchronous muscles (Josephson, 1985a).

The work loop technique has proved to be an extremely useful and versatile experimental tool, enabling physiologists and biomechanists to gain insight into: the principles of how muscles function (Ahn and Full, 2002; Askew and Marsh, 1998; Askew et al., 1997; Holt et al., 2014; James et al., 1996); how muscles respond dynamically to perturbations (Gordon et al., 2020; Rice et al., 2023; Schwaner et al., 2024); muscle functional changes in relation to pathophysiology (Espino-Gonzalez et al., 2021; Warren et al., 2020); and muscles' mechanical function during locomotion (Altringham et al., 1993; Askew and Marsh, 2001; Josephson, 1985b; Marsh and Olson, 1994). Other studies have used sinusoidal cycles and optimised stimulation parameters to investigate the maximum performance of a muscle under cyclical conditions (Askew and Marsh, 1997; Kissane et al., 2018). This approach may not reflect a muscle's *in vivo* function but provides a standardised approach to enable muscle properties to be compared. Consequently, using this approach, muscle performance can be compared between muscles (both inter- and intra-specifically) under these standard conditions, and the cycle frequency at which maximum net power is generated can be determined and related back to *in vivo* muscle function (Bahlman et al., 2020). In scenarios where the research question relates specifically to how a muscle functions *in vivo*, the work loop technique may be used to impose muscle length trajectories that have been recorded *in vivo*, and phasic stimulation to match the onset and offset times of *in vivo* activity [determined from simultaneously recorded electromyography (EMG)] (Ahn and Full, 2002; Askew and Marsh, 2001; Bemis and Nishikawa, 2023; Girgenrath and Marsh, 1997; Marsh and Olson, 1994; Marsh et al., 1992; Rice et al., 2023).

This latter approach relies on having both *in vivo* recordings of muscle length change and muscle EMG activity to assess *in vivo* muscle performance, as both these inputs have substantial implications for work loop estimates of performance (Ahn and Full, 2002). Having direct measures of muscle length trajectory offers the opportunity to expose muscles to temporally appropriate lengthening and shortening velocities, which are known to affect the force-generating capacity of a muscle and consequently the power produced (Askew and Marsh, 1997, 1998). These data are typically acquired using sonomicrometry (Ross et al., 2024), fluoromicrometry (Camp et al., 2016) or magnetomicrometry (Taylor et al., 2021) and provide unequivocal readouts of fibre length changes. Equally, EMG-derived stimulation duration and phase are known to significantly alter predictions of muscle performance (Altringham and Johnston, 1990; Josephson, 1985a; Rice et al., 2023). Muscle onset and offset are easily derived from muscle EMG; however, muscle activation is a graded response, and this variation in muscle activation intensity has not been accounted for in the supramaximal ‘square-wave’ stimulation protocols that have typically been used. This may be important in studies where the aim is specifically to understand muscle function *in vivo*, because the rates of force development and relaxation, which are themselves important determinants of power and work production (Caiozzo and Baldwin, 1997; Josephson, 1993; Rice et al., 2023), are affected by the pattern of activation. Recent development of muscle stimulators capable of modulating voltage/current output (Bukovec et al., 2020) provides an opportunity to emulate more representative muscle activation patterns, where, for example, EMG may be used as a surrogate (Roberts and Gabaldón, 2008) to drive graded recruitment that may provide better estimates of muscle *in vivo* function and performance.

Here, we set out to investigate the importance of muscle activation pattern on estimates of muscle performance during cyclical contractions. To do this, we used data derived from two different experimental approaches. The first used *in vivo* recordings of fibre length change (from fluoromicrometry) and activation patterns (from recorded EMG) in the digastric muscle of rabbits during feeding (Kissane et al., 2024). This multifunctional muscle (Ahn and Full, 2002) acts as a motor, generating positive work to depress and open the jaw, while also functioning to stabilise the jaw by producing negative work during jaw opening. The measured *in vivo* EMG intensity was replayed back onto the muscle *in situ* to replicate *in vivo* activation while subjecting the muscle to *in vivo* fibre strains. This was compared with the supra-maximal square-wave activation pattern that has routinely been used in work loop studies. The second approach used fibre strains and activation patterns predicted by a musculoskeletal model of mouse trotting (Charles et al., 2018, 2024). Musculoskeletal models are a widely used method to predict muscle functional outputs (Wakeling et al., 2021), which can help unravel dynamic changes in muscle function in response to pathophysiological remodelling (Seth et al., 2018) and can provide insight into how muscles may be functioning in species that have yet to be quantified (e.g. mice; Charles et al., 2018) or in which it is technically difficult to do so (e.g. humans; Charles et al., 2020). Here, we used the model-derived estimates of fibre length change and muscle recruitment for the soleus (SOL) and extensor digitorum longus (EDL) muscles and replayed these *ex vivo* onto the muscle. Again, these measurements were compared with the supra-maximal square-wave activation pattern that has routinely been used in work loop studies. In addition, the mouse SOL and EDL were subjected to sinusoidal work loops optimised for maximal net power, to enable comparison between the predicted *in vivo* functional protocols and those commonly used to assess the power-generating capacity of muscles (Shelley et al., 2024).

## MATERIALS AND METHODS

All experimental procedures were performed in accordance with the UK Animals (Scientific Procedures) Act 1986 and approved by the University of Leeds Animal Welfare and Ethical Review Committee. This work conforms to the ethical requirements outlined by the journal, and is presented in accordance with guidelines for animal work (Percie du Sert et al., 2020).

### Animals

Four male New Zealand white rabbits (Envigo) (2388±95 g) and 11 in-house male C57B6 mice (25.29±2.04 g) were used in this study. Animals were housed under a 12 h:12 h light:dark cycle at 21°C and had *ad libitum* access to food and water.

### *In situ* rabbit preparation

Rabbits were anaesthetised with a s.c. injection of ketamine (Ketavet, Zoetis; 50 mg kg^−1^) and xylazine (Rompun, Bayer; 5 mg kg^−1^). Following confirmation of anaesthesia, an i.v. canula was implanted to deliver ketamine and xylazine throughout the remainder of the experiment. The digastric muscle was exposed via an incision running the length of the dorsal aspect of the jaw. A 3 mm hole was drilled through the mandible and a custom 3D printed mould was screwed to the bone and attached to a custom rig to allow for muscle length to be changed. The distal end of the digastric muscle was sutured to a stainless-steel loop with silk suture (3-0, LOOK Braided suture) and attached to the ergometer (305B-LR; Aurora Scientific Inc., London, ON, Canada) via a stainless steel connecting rod. Parallel platinum wires (0.4 mm diameter) were implanted into the digastric muscle using a 25-gauge needle and sutured into place. The rabbit was left for 30 min to recover from electrode implantation, prior to the beginning the muscle mechanical experiments. Throughout the experiment, the rabbit's body temperature was maintained at 37°C (Animal Temperature Controller 2000, WPI) and the muscle was regularly irrigated with warmed saline. Following completion of the experiment, rabbits were culled with an overdose of pentobarbital.

### *Ex vivo* mouse muscle preparation

Mice were culled using approved Schedule 1 methods. The hindlimb of the mouse was transferred to chilled (4°C), oxygenated (95% O_2_, 5% CO_2_) Krebs–Henseleit solution (mmol l^−1^: 117 NaCl, 4.7 KCl, 2.5 CaCl_2_, 1.2 MgSO_4_, 24.8 NaHCO_3_, 1.2 KH_2_PO_4_ and 11.1 glucose; Burton, 1975). The whole SOL and EDL were dissected free and aluminium foil clips were attached to the proximal and distal ends of the muscles (Askew and Marsh, 1997). The SOL and EDL were suspended vertically in a flow-through Perspex chamber filled with circulating, oxygenated Krebs–Henseleit solution at 37±0.5°C. Muscles were attached to an ergometer (series 300B-LR; Aurora Scientific Inc.) via a lightweight stainless-steel rod; the position of the ergometer and therefore the length of the muscle could be controlled using a digital height gauge (Mitutoyo Corporation, Kanagawa, Japan). Muscles were left for 30 min to thermoequilibrate (Askew and Marsh, 1997; Kissane et al., 2022; Kissane and Askew, 2024) and recover from the dissection. Parallel platinum electrodes were placed inside the chamber on either side of and parallel to the muscle.

### Muscle work loop experiments

**Fig. 1.**
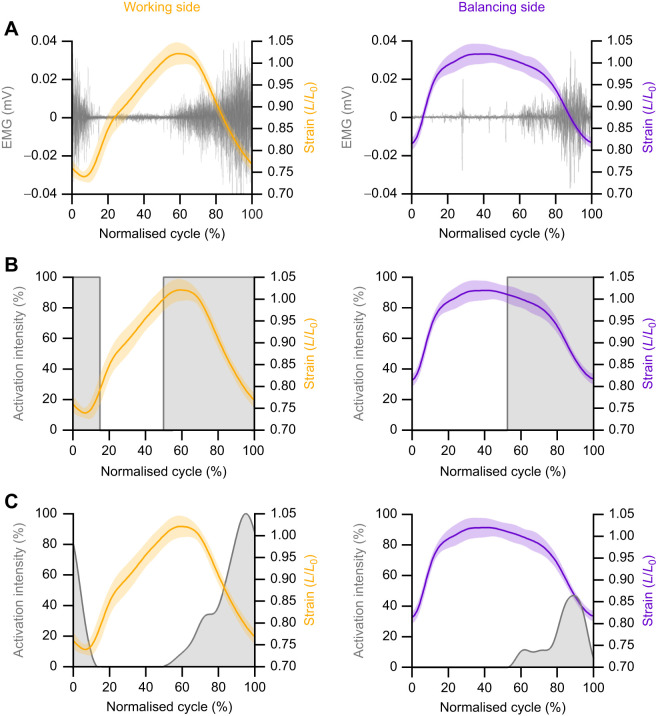
**Stimulation paradigms for the rabbit digastric muscle using the work loop technique.** Typically, to replay muscle electrography (EMG) and length trajectories onto a muscle (A; raw data), the onset and offset of the EMG are determined, and a square-wave maximal activation is used, defined by EMG timings (B). However, muscles typically follow a graded activation, and the importance of accounting for the level of activation on muscle mechanics has yet to be determined. Here, in our rabbit experiments, we compared square-wave activation (B) with an EMG envelope that incorporates graded muscle intensity of activation (C). Notably, in addition to the differences in strain trajectory of the digastric muscle when functioning on the working side (orange) compared with the balancing side (purple), the muscle activation levels also significantly differ (C). *L*_0_, length at which muscle generated maximum twitch force.

**Fig. 2.**
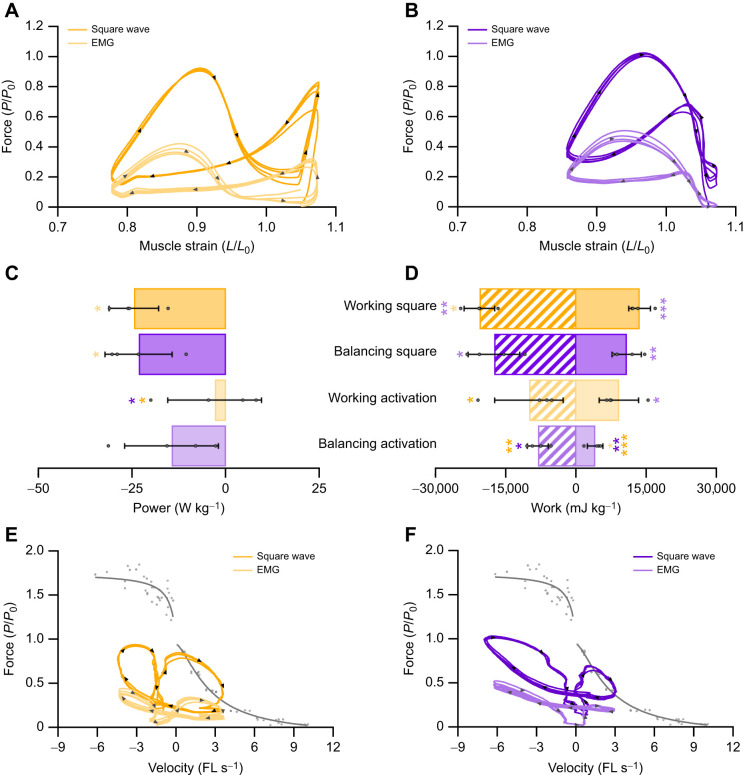
**The implications of muscle activity on estimated mechanical function in the digastric muscle.** The digastric muscle was differentially activated using either traditional square-wave activation or EMG-derived activation curves. Muscle work loops (four repeated cycles from one individual) are presented for the digastric muscle when differentially activated under working-side strain (A) and balancing-side strain (B). The absolute net power generated by the muscle is dependent on the level of activation (C). The level of muscle activation has a significant impact on the net work done by the muscle (D). Overlaying work loops onto the force–velocity curve (four repeated cycles from one individual) for the digastric muscle highlights the overestimation of force when using square-wave activation for working-side (E) and balancing-side (F) muscle mechanics. FL, fibre length. Data (*n*=4) are means±s.d. A one-way ANOVA was used to determine significant differences among groups with *post hoc* comparisons made using Fisher's Least Significant Difference test (*P<0.05, **P<0.01 and ***P<0.001).

All muscles were subjected to a series of isometric twitches (supramaximal stimulus of 0.2 ms pulse) and muscle lengths were incrementally increased to find the optimal length for maximum force generation (*L*_0_) which was used as the starting length in both the rabbit and mouse muscles (Figs 1 and 2; Fig. S1). Stimulation during the isometric tetanus and work loop stimulations was delivered at 200 Hz for the digastric, 150 Hz for the SOL and 250 Hz for the EDL muscles (Kissane and Askew, 2024).

The work loop technique (Josephson, 1985a) was used to quantify force and power generation over the course of the cyclical length change in the rabbit digastric muscle (cycle duration of 0.275 ms) (Kissane et al., 2024), and mouse SOL and EDL muscles (cycle duration of 0.128 ms) (Charles et al., 2018). The strain trajectory and activity patterns of the rabbit digastric muscle were determined previously (Kissane et al., 2024) during feeding on pellets for two conditions: where the muscle operated on the working-side or on the balancing-side of the jaw (Fig. 1). The fibre strain trajectory and activity patterns of the mouse SOL and EDL muscles were estimated using a computational simulation model of trotting (Charles et al., 2018) (Fig. S1). The strain trajectories were imposed on the muscles using a muscle ergometer (model 300B-LR for SOL and EDL and model 305B-LR for the rabbit digastric) and activity patterns delivered to the muscle via a custom-built stimulator (modified 701C stimulator, Aurora Scientific Inc.), with both devices being controlled using custom-written protocols (Dynamic Muscle Control software, Aurora Scientific Inc.).

Here, we used two stimulation protocols: (i) a supramaximal square-wave pattern of stimulation and (ii) a graded *in vivo* activation pattern. The supra-maximal square-wave activation pattern was delivered at timings that corresponded to the *in vivo* onset and offset times of the EMG relative to the muscle's length trajectory (Fig. 1A,B). The *in vivo* graded activation pattern was based on the intensity of activation determined from the recorded EMG (rabbit digastric) or model-derived relative activity (mouse SOL and EDL) (Fig. 1C), using a modified version of the protocol used by Bukovec et al. (2020). For each muscle, an isometric twitch force–current recruitment curve was generated to define the upper and lower current thresholds of the muscle, i.e. the lowest current that yielded maximal twitch force (upper threshold) and the lowest current that resulted in force generation (lower threshold) (Charles et al., 2024; Kissane et al., 2024). These thresholds were used to determine the stimulation currents for the square-wave activation (Fig. 1B; Fig. S1A) and the graded patterns of activation (Fig. 1C; Fig. S1B,C).

For the rabbit digastric muscle, the variable stimulation patterns were derived from muscle EMG, where the signals were rectified and a 30 Hz low-pass filter was applied to create a relative activity envelope (Fig. 1C) (Roberts and Gabaldón, 2008). The recruitment intensity was determined as the ratio of the integrated EMG relative to the working side, i.e. working side (100% recruitment; Fig. 1C, orange trace) and balancing side (47% recruitment; Fig. 1C, purple trace) (Kissane et al., 2024). The timings (onset and offset) of muscle activity relative to the strain trajectory were identified when voltages exceeded a threshold of two standard deviations above the baseline EMG signal (Roberts and Gabaldón, 2008). For the rabbit digastric muscle, either the activation was ‘square-wave’ supra-maximal activation (Fig. 1B) or the intensity of activation was modulated based on EMG, produced by the filtered activity envelope (Fig. 1C). Therefore, muscles underwent four different, randomised work loop conditions: square-wave activation under working- and balancing-side strain trajectories, and representative *in vivo* activation stimulation based on their integrated EMG levels for working- and balancing-side strain trajectories. Following the identification of the length at which the muscle generated maximum twitch force (*L*_0_), the digastric muscle underwent a control tetanus to determine a baseline isometric force to assess and account for any decline in the preparation. Subsequently, three randomised work loop trials with each of the stimulation protocols were delivered, each trial comprising five repeated cycles (with an initial passive work loop followed by four active work loops), oscillating about *L*_0_, with 5 min rest between each. This was followed by another control tetanus. This was repeated until all stimulation protocols were completed, or until the tetanic force had declined below 70% of the initial value.

The OpenSim musculoskeletal model of mouse trotting outputs a muscle activation curve equivalent to the relative activity envelop we generated from muscle EMG (Fig. S1); therefore, the current recruitment upper and lower thresholds could be applied to these curves directly to generate the variable stimulation wave. The SOL and EDL were subjected to muscle length trajectories and activity patterns that matched modelled *in vivo* muscle behaviour (Charles et al., 2018) (Fig. S1) as well as sinusoidal work loops with strain and stimulation parameters optimised to maximise net power output (Askew and Marsh, 1997). The SOL underwent five randomised stimulation conditions: optimised 5 Hz and 8 Hz sinusoidal work loops with supra-maximal, square-wave activation (Askew and Marsh, 1997), which were cycled around *L*_0_, and ‘square-wave’, ‘100% activation’ and muscle-level ‘activation’ stimulation under trotting-derived strain trajectories (Charles et al., 2018), which were strains derived relative to optimised muscle length (Fig. S1). The EDL underwent four randomised stimulation conditions: optimised 8 Hz sinusoidal work loops with supra-maximal, square-wave activation cycled around *L*_0_, ‘square-wave’, ‘100% activation’ and muscle-level ‘activation’ stimulation under trotting-derived strain amplitudes relative to optimised muscle length (Fig. S1). Following the identification of *L*_0_, the SOL and EDL underwent a control tetanus to determine a baseline isometric force to assess and account for the decline in preparation, after which three randomised work loop stimulation paradigms were delivered, consisting of five repeated cycles (with an initial passive work loop followed by four active work loops) with 5 min rest between each. This was followed by another control tetanus. This was repeated until all stimulation paradigms were completed, or until the preparation had declined below 70% of the initial tetanic force.

### Statistics

Statistical differences were analysed in SPSS 28 (28.0.1.1, IBM). A one-way ANOVA was used to determine significant differences among stimulation paradigms across the individual muscles. The digastric dependent variables (net power, positive and negative work) each had four between-subject factors: ‘working square-wave’, ‘working activation’, ‘balancing square-wave’ and ‘balancing activation’. Between-subject factors for the SOL dependent variable net power include: ‘5Hz’, ‘8Hz’, ‘square-wave’, ‘100% activation’ and ‘activation’. The positive and negative work had only three between-subject factors: ‘square-wave’, ‘100% activation’ and ‘activation’. Similarly, the between-subject factors for the EDL-dependent variable net power include: ‘8Hz’, ‘square-wave’, ‘100% activation’ and ‘activation’. Again, the positive and negative work had only three between-subject factors: ‘square-wave’, ‘100% activation’ and ‘activation’. Given the low sample number in the digastric experiments, all *post hoc* comparisons were calculated using Fisher's Least Significant Difference test. The threshold for statistical significance was set to *P*<0.05. All data processing and figures were plotted using Igor Pro 8 (v8.0.4.2, Wavemetrics, Portland, OR, USA) with data presented as means±s.d.

## RESULTS

### Rabbit digastric muscle performance

**Fig. 3.**
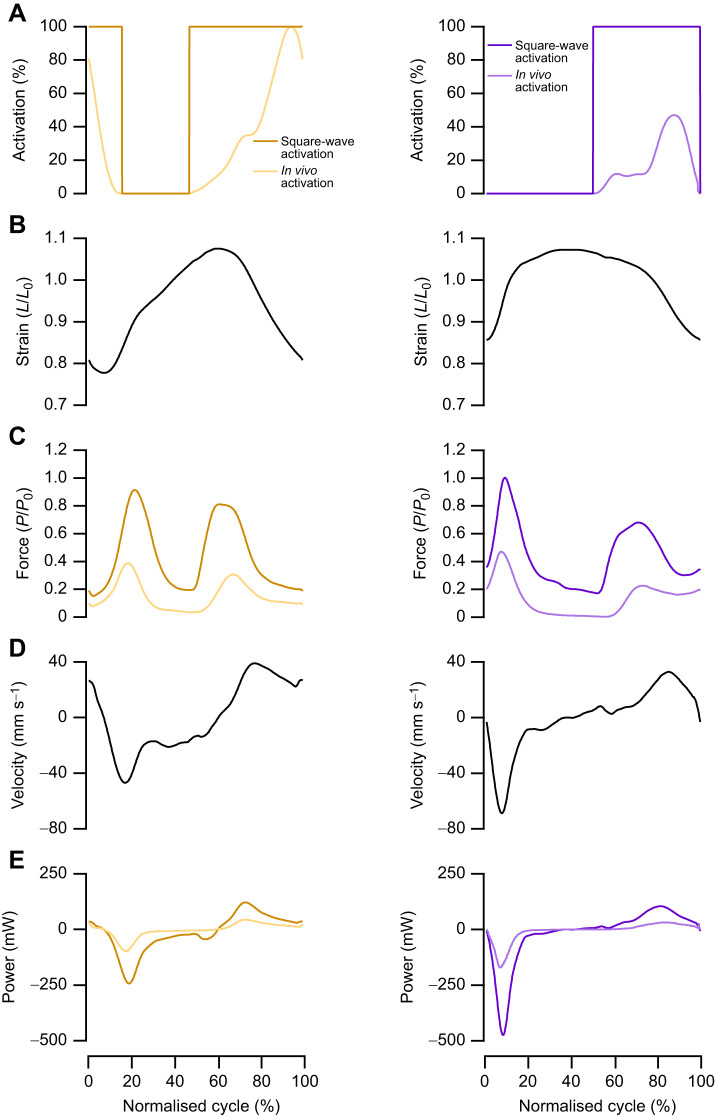
**Mechanical properties of the rabbit digastric muscle across activation paradigms.** Time-normalised data for a single cycle (275 ms) are presented for the working side (left) and balancing side (right). The representative stimulation activation parameters (A) and strain trajectories (B) played onto the muscles are shown. The subsequent force generated by the muscle (C) highlights that square-wave activation leads to greater forces throughout the masticatory cycle when functioning on either the working or balancing side, when compared with representative activation. The distinct strain trajectories across the two feeding behaviours undergo different muscle fibre velocities (D). Consequently, the greater forces experienced during square-wave activation led to higher peak positive and negative power (E).

Estimates of muscle power were significantly impacted by muscle activation pattern (*F*_3,12_=3.630, *P*=0.045; Figs 2A–C and 3); specifically, estimates of muscle net power were lower for the working square-wave activation compared with the replayed EMG activation levels (−24.56±6.57 versus −3.04±12.51 W kg^−1^, *P*=0.013). Consequently, there were significant effects of muscle activation pattern on estimates of positive (*F*_3,12_=7.257, *P*=0.005; Figs 2D and 3E) and negative work (*F*_3,12_=5.677, *P*=0.12; Figs 2D and 3E) for both working- and balancing-side strain trajectories. Namely, the major differences are that square-wave activation generated significantly greater negative work for the working-side (−20,592±3230 versus −10,027±7325 mJ kg^−1^, *P*=0.011) and balancing-side (−17,525±5485 versus −8166±2258 mJ kg^−1^, *P*=0.021) muscle length trajectories, compared with the graded *in vivo* activation pattern, while square-wave activation only generated significantly greater positive work on the balancing side (10,779±3094 versus 4030±1633 mJ kg^−1^, *P*=0.007). The implications for muscle activation level and length trajectory on muscle work are presented in the force–velocity relationship for the digastric muscle (Fig. 2E,F). Specifically, square-wave stimulation of the muscle led to activation long into muscle lengthening (Fig. 3A,B), which resulted in rapid eccentric activations (Fig. 3C). The consequence of this appears to be an enhanced force generated by the muscle during shortening that exceeds forces predicted by the isotonic force–velocity relationship.

### Mouse soleus and EDL muscle performance

**Fig. 4.**
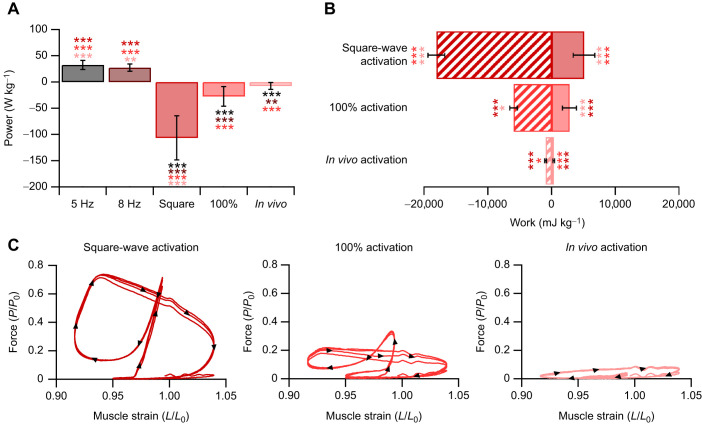
**The implications of muscle activity on estimated mechanical function in the mouse soleus.** There is a significant impact of muscle activation and length trajectories on the net power generated by the soleus (A). The soleus primarily performs negative work during trotting, which the sinusoidal 5 Hz and 8 Hz length trajectories do not appropriately emulate. Consequently, the square-wave maximum activation and 100% activation significantly overestimate the negative work generated by the muscle (B). Representative work loops (four repeated cycles from one individual) for the supra-maximal square-wave activation, 100% *in vivo* activation pattern and *in vivo* activation pattern adjusted for activation intensity (C). Data (*n*=8) are means±s.d., with a one-way ANOVA used to determine significant differences among groups. *Post hoc* comparisons were made using Fisher's Least Significant Difference test (*P<0.05, **P<0.01 and ***P<0.001).

There was a significant difference in muscle net power (*F*_4,35_=54.687, *P*<0.001; Fig. 4A), negative work (*F*_2,21_=32.034, *P*<0.001; Fig. 4B) and positive work (*F*_2,21_=32.876, *P*<0.001; Fig. 4B) performed by the SOL depending on the work loop parameters used. Across the square-wave activation, 100% activation and *in vivo*-level activation, the SOL generates net negative power, as evidenced by the work loops (Fig. 4C). Yet the magnitude of negative power, and consequently negative work and positive work differed significantly depending on the activation level, with the square-wave activation producing 19-fold more negative work (−18,061±6889 mJ kg^−1^ versus −965±440 mJ kg^−1^, *P*<0.001; Fig. 4B) and 16-fold more positive work (5060±1707 mJ kg^−1^ versus 314±144 mJ kg^−1^, *P*<0.001; Fig. 4B).

**Fig. 5.**
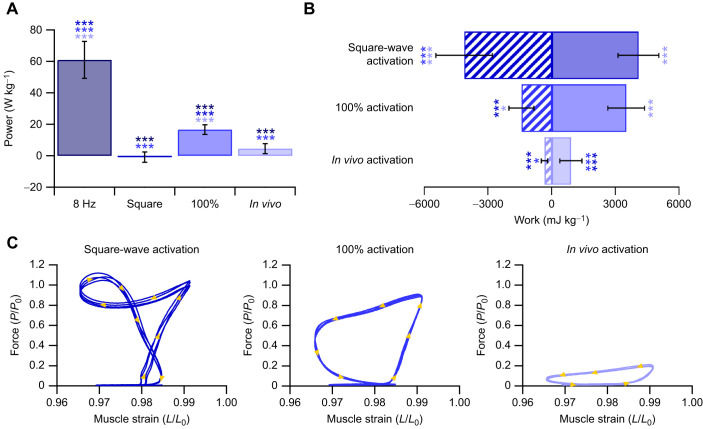
**The implications of muscle activity on estimated mechanical function in the mouse extensor digitorum longus (EDL).** There is a significant impact of muscle activation and length trajectories on the power generated by the EDL (A). The EDL primarily performs positive work during trotting, which the sinusoidal 8 Hz length trajectory significantly overestimates. Consequently, the square-wave and 100% activation significantly overestimate the negative and positive work generated by the muscle (B). Representative work loops (four repeated cycles from one individual) for the square-wave, 100% activation and *in vivo* muscle activation (C); of note, the square-wave activation during muscle lengthening means that the muscle generates forces greater than maximum isometric tetanic force. Data (*n*=9) are means±s.d., with a one-way ANOVA used to determine significant differences among groups. *Post hoc* comparisons were made using Fisher's Least Significant Difference test (*P<0.05 and ***P<0.001).

**Fig. 6.**
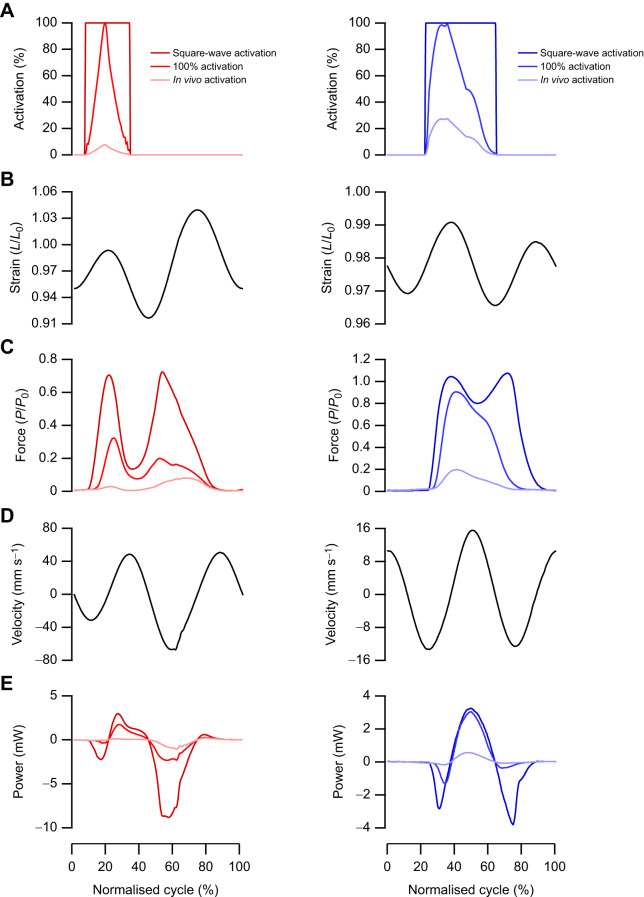
**Mechanical properties of the mouse soleus and EDL.** Time-normalised data for a single cycle (128 ms) for the soleus (SOL; left) and EDL (right). The representative activation parameters (A) and strain trajectories (B) played onto the muscles are shown. The subsequent force generated by the muscle (C) highlights that square-wave activation and 100% activation led to greater forces during muscle shortening and encroached into portions of muscle lengthening, leading to large eccentric force spikes for both the SOL and EDL. The subsequent velocity traces in response to the individual muscle length trajectories (D). Consequently, the greater forces experienced during square-wave activation and 100% activation led to higher peak positive and negative power (E) across both the SOL and EDL.

Similar to the SOL, there were significant differences in muscle net power (*F*_3,32_=169.73, *P*<0.001; Fig. 5A), negative work (*F*_2,24_=47.542, *P*<0.001; Fig. 5B) and positive work (*F*_2,24_=40.196, *P*<0.001; Fig. 5B) performed by the EDL depending on the work loop parameters used (Figs 5 and 6). With square-wave activation, the EDL undergoes a large portion of active muscle lengthening (Figs 5C and 6A–C) generating force above *P*_0_, while the negative work done on the muscle cancels out the positive work during shortening, resulting in a net power of −0.87±3.24 W kg^−1^. Using the 100% activation paradigm, the EDL generated a large anti-clockwise (positive) work loop (Fig. 5B,C), with the positive and negative work significantly higher than those generated when using the graded *in vivo* activation pattern. The magnitude of negative and positive work differed significantly by 12-fold (−4122±1335 mJ kg^−1^ versus −340±147 mJ kg^−1^, *P*<0.001; Fig. 5B) and 5-fold (4083±957 mJ kg^−1^ versus 902±514 mJ kg^−1^, *P*<0.001; Fig. 5B) between square-wave activation and the graded activation levels, respectively.

**Fig. 7.**
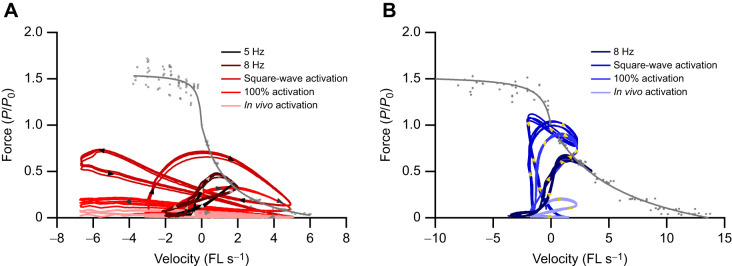
**Mouse power trajectories.** Fibre force and velocity from each work loop trajectory for the soleus (A) and EDL (B). Data are plotted onto the complete concentric and eccentric force–velocity relationship for each muscle.

The optimum cycle frequency for maximum net power generation in the mouse SOL during sinusoidal length trajectories is 5 Hz, at which a net power of 32.4±8.7 W kg^−1^ was generated. However, mice typically trot with a cycle frequency of 8 Hz, and when optimised sinusoidal work loops at 8 Hz were performed, the net power was not significantly different to that generated at 5 Hz (27.3±7.07 W kg^−1^, *P*=0.633; Fig. 4A). The optimum cycle frequency for maximum net power generation in the mouse EDL is ∼8 Hz, aligning with the trotting gait cycle frequency. The power generated by an optimised sinusoidal work loop at 8 Hz was 61.0±11.7 W kg^−1^ (Fig. 5A). Muscle force–velocity curves are presented for the SOL (Fig. 7A) and EDL (Fig. 7B) with overlaid force–velocity profiles during the work loops for the square-wave, 100% activation and *in vivo* activation, as well as at 5 Hz (SOL only) and 8 Hz (SOL and EDL). Across both the SOL and EDL, square-wave activation generated a large peak in eccentric force (Fig. 6C,D), which continued into the initiation of muscle shortening. This elevated force spill-over leads to an instance where both the SOL and EDL generate greater forces than predicted by isotonic muscle shortening (Fig. 7).

## DISCUSSION

To better understand how muscles function *in vivo*, physiologists have utilised the work loop technique (Josephson, 1985a) to simulate muscle behaviour*,* playing back muscle length trajectories onto the muscle and stimulating the muscle (supra-maximally) at times corresponding to the period of EMG activity (Altringham et al., 1993; Askew and Marsh, 2001; Marsh and Olson, 1994; Marsh et al., 1992). This approach offers the opportunity to expose muscles to temporally appropriate length excursions and consequently physiologically meaningful shortening and lengthening velocities, which are known to have dramatic implications for the magnitude and timing of force generation by the muscle, and consequently for the power produced (Askew and Marsh, 1997, 1998). While this approach can successfully simulate muscle length change, the pattern of activation does not reflect the variation in the intensity of muscle activity that occurs *in vivo* during the period of measured EMG activity. Here, we show that estimates of muscle performance during physiologically representative length excursions are significantly different when muscles are stimulated using traditional, supra-maximal square-wave activation compared with when graded *in vivo* patterns of activation are used.

The work loop technique is a highly versatile approach for assessing muscle function (Sponberg et al., 2023), with input length and activation parameters modifiable to explore a range of biological questions. The sensitivity of the work loop approach to strain trajectories (Askew and Marsh, 1997), starting length on the force–length relationship (Shelley et al., 2024), phase of stimulation (Ahn and Full, 2002; Sawicki et al., 2015) and stimulation frequency (Stevens, 1996) brings with it inherent uncertainties in the translation to and interpretation of *in vivo* function. However, careful consideration and control of these variables allows for meaningful interpretations to be made. The activation of muscles during the work loop is typically modified by altering stimulation phase (Ahn and Full, 2002) and frequency (Stevens, 1996), which can be manipulated to closely match *in vivo* forces measured from the combined tendon of several muscles (Bemis and Nishikawa, 2023; Rice et al., 2023). Yet, modification of these two parameters does not easily allow for replication of the gradual and often submaximal activation seen *in vivo*. Therefore, the development of muscle stimulators capable of modulating voltage/current output (Bukovec et al., 2020) brings a unique opportunity to more closely represent muscle activation patterns seen *in vivo*, the rationale being that the strong correlation with *in vivo* force and EMG (Gabaldón et al., 2008; Hedrick et al., 2003) allows for the rectified EMG envelop to be used as an output to drive stimulation of a muscle.

In both the rabbit digastric and mouse hindlimb muscles, square-wave activation resulted in significantly greater estimates of power, and positive and negative work. In the context of the rabbit digastric muscle, the most notable difference was that square-wave activation resulted in an 8-fold greater estimate of negative power when functioning on the working side during feeding. The sustained supramaximal activation during lengthening resulted in greater forces (Fig. 3A–C), probably due to the rapid phase 1 component present during eccentric muscle activation (Kissane and Askew, 2024), which led to a greater negative instantaneous power (Fig. 3E). This difference in estimates of muscle performance has meaningful implications for the subsequent interpretations of how this muscle functions *in vivo*. Using square-wave activation, the digastric muscle would appear to absorb more energy than the work generated, whereas with the graded *in vivo* activation pattern, the energy absorbed and work generated are in fact similar in magnitude. Similarly, in the hindlimb muscles of the mouse, there was a substantial difference in muscle performance when muscles were subjected to a graded *in vivo* activation pattern, compared with the traditional supramaximal square-wave activation. Square-wave activation in the SOL led to a 14-fold greater estimate of muscle power compared with that of the muscle activation (Figs 4 and 6), while in the EDL, square-wave activation did not produce a significantly different net power to that of the graded *in vivo* activation pattern (Figs 5 and 6). However, the square-wave activation of the EDL resulted in a 12-fold greater amount of negative work compared with that during the graded *in vivo* activation pattern. These data cumulatively highlight the importance of muscle activation level on estimates of muscle performance, and in the context of experiments designed to uncover how a muscle functions *in vivo*, the stimulation paradigm must attempt to better replication muscle activation.

Many experiments have been carried out using the work loop technique to determine the limits to muscle power generation (Askew, 2023), using sinusoidal length changes with the timing and duration of supramaximal stimulation optimised for maximal net power output. Such experiments allow muscle performance to be compared across a range of cycle frequencies and strains, both inter- and intra-specifically under standardised conditions, allowing a muscle's potential contribution to locomotion to be determined. Furthermore, this standardised approach has become a valuable tool in the assessment of pathophysiological or adaptive remodelling of skeletal muscle; for example, in relation to ageing (Tallis et al., 2014), spinal cord injury (Warren et al., 2020), heart failure (Espino-Gonzalez et al., 2021) and assessment of cardiac muscle function (Fletcher et al., 2020; Layland et al., 1995a,b). However, we demonstrate that, in the case of the SOL, these optimised work loops can differ substantially from those determined under simulated *in vivo* conditions (Fig. 4). Therefore, extrapolating the findings from this type of experiment to *in vivo* performance should be done with caution.

### Limitations

In the rabbit digastric muscle, we were unable to relate the directly measured *in vivo* fibre lengths to the length of muscle *in situ*; instead, the mid-length of the muscle was set such that during the cyclical contractions, the maximum length of the muscle coincided with the plateau of the length–force relationship and the muscle therefore operated on the plateau and ascending limb of the length–force relationship. Recent work has shown that power output during cyclical contractions in the mouse EDL and soleus muscles is insensitive to starting length between starting lengths of 0.95 to 1.1 *L*_0_ and 0.90 to 1 *L*_0_, respectively (Shelley et al., 2024). Furthermore, the descending limb of the length–force relationship is inherently unstable (Morgan, 1990) and therefore unlikely to represent lengths used *in vivo*. While we cannot be certain that the operating lengths used *in situ* reflect those *in vivo*, we think it unlikely that our selection of starting length affected our assessment of muscle force. Here, we have demonstrated that the time course and magnitude of force generation differ between our stimulation protocols. As *in vivo* muscle force has not been measured, we cannot be certain that our stimulation protocol based on graded *in vivo* activation replicates *in vivo* force. True validation of this approach must wait until such data are available.

### Conclusion

In conclusion, we have demonstrated that there is a considerable difference in estimating muscle performance using *in vivo-*derived graded activation patterns, compared with the traditionally used supramaximal square-wave activation pattern. These findings suggest that where the goal of the research is to understand the time course and magnitude of force and power generation, e.g. for understanding muscle function *in vivo* or for validating musculoskeletal models, both the time course and intensity of activation should be accurately represented.

## Supplementary Material

10.1242/jexbio.248051_sup1 Supplementary information
