# Supplementary material for: The importance of muscle activation on the interpretation of muscle mechanical performance
Source: J Exp Biol. 2024 Nov 8;227(21):jeb248051. doi: 10.1242/jeb.248051 (PMC11574351; doi:10.1242/jeb.248051)
Supplement: Supplementary information [file jexbio-227-248051-s1.pdf]

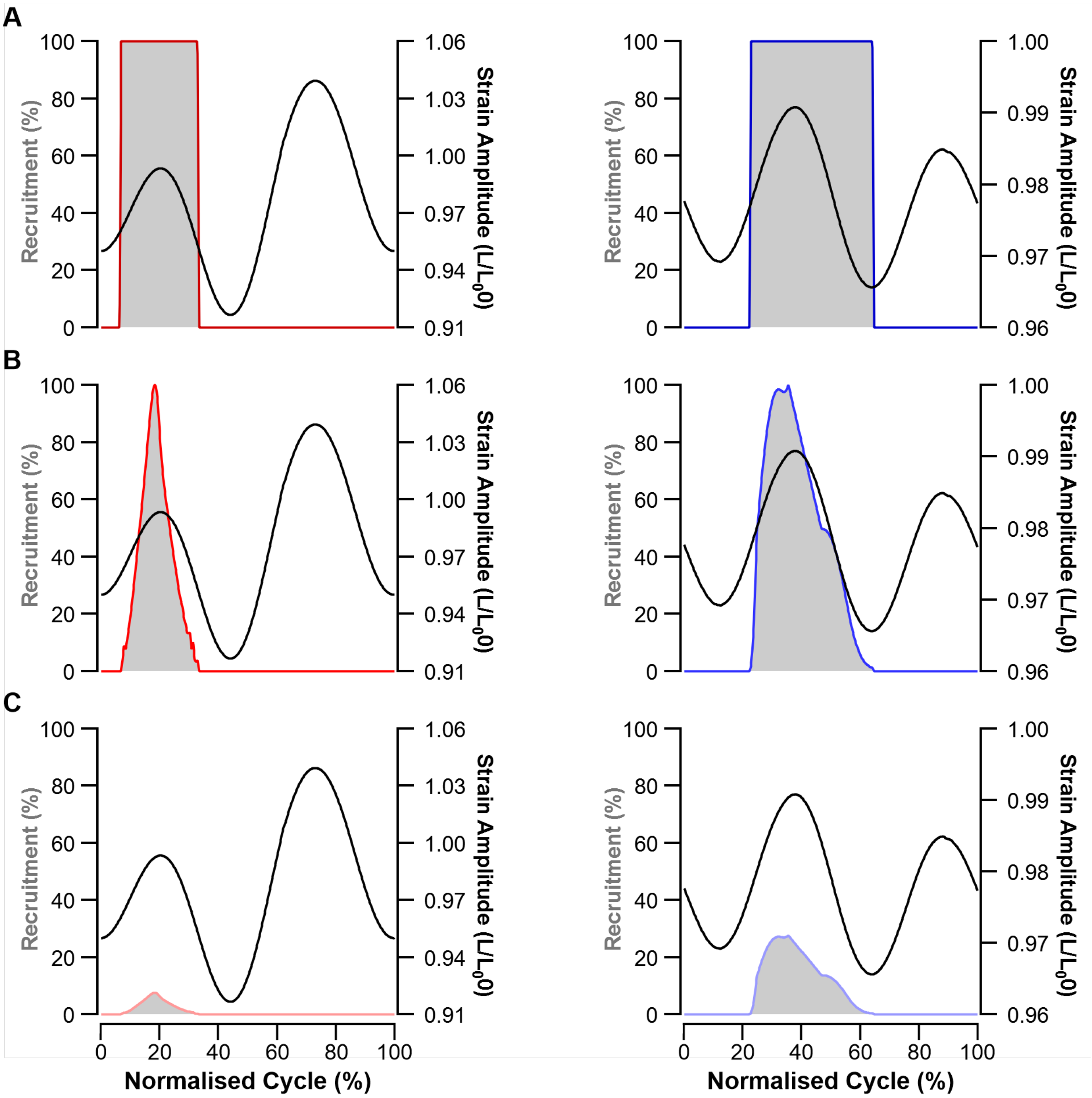

Fig. S1. Stimulation paradigms for the work loop technique in the mouse. Soleus (SOL) (left column) and extensor digitorum longus (EDL) (right column) strain amplitudes were derived from a musculoskeletal model of mouse trotting with a stride frequency of 0.128ms. Here we have assessed the effect of muscle activation level on the mechanical performance of the SOL and EDL using square-wave activation (A), 100% activation curves (B) and the estimated in vivo activation levels (C) from the Hill-type model.

Journal of Experimental Biology • Supplementary information
